# Supplementary material for: Bento: a toolkit for subcellular analysis of spatial transcriptomics data
Source: Genome Biol. 2024 Apr 2;25:82. doi: 10.1186/s13059-024-03217-7 (PMC11289963; doi:10.1186/s13059-024-03217-7)
Supplement: Supplementary file 1 — Additional file 1: Supplementary Figures and Table S1. Supplementary figures and machine learning classifier feature descriptions. [file 13059_2024_3217_MOESM1_ESM.pdf]

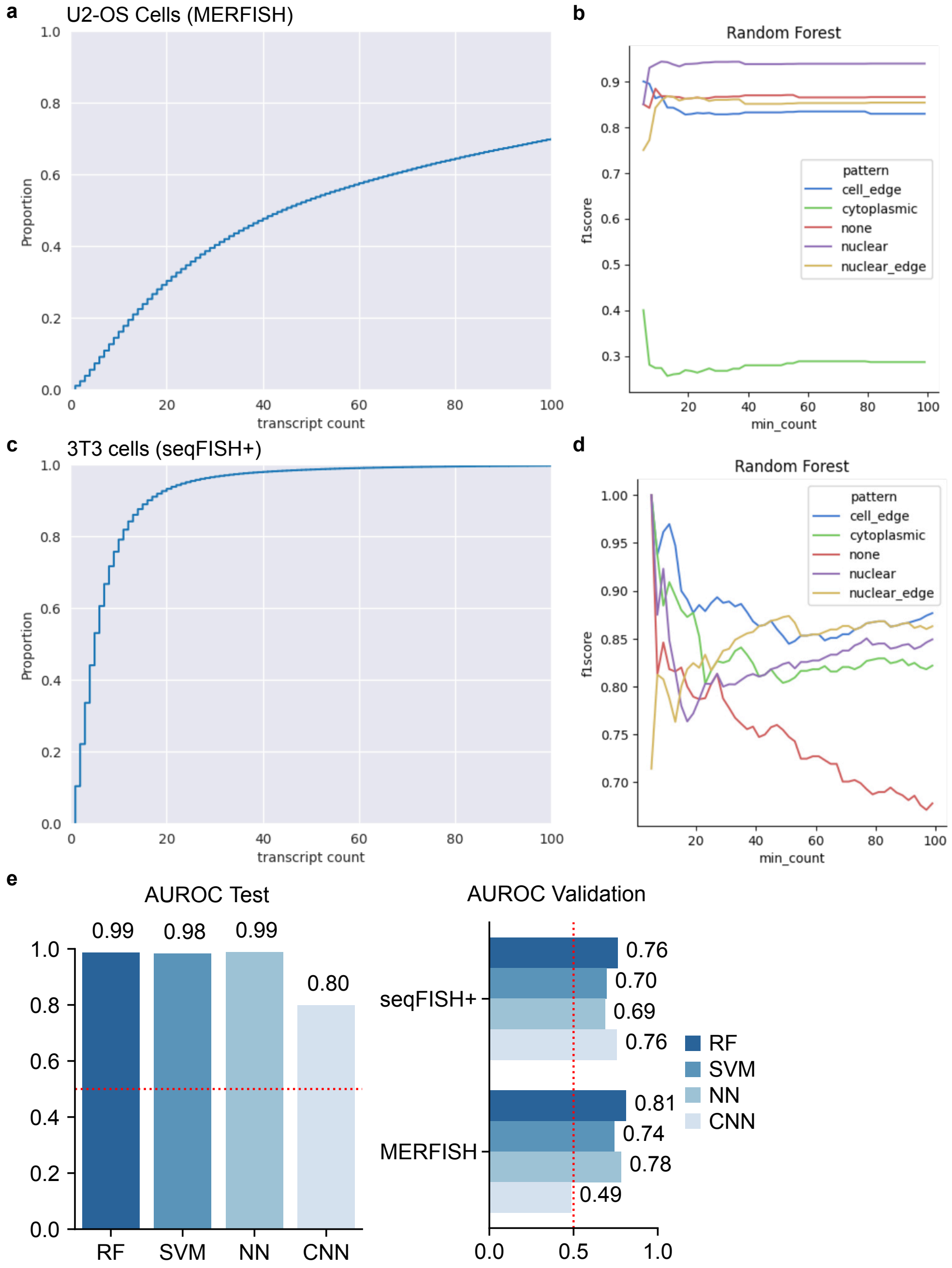

a
U2-OS Cells (MERFISH)
Proportion
transcript count
b
Random Forest
f1score
min_count
pattern
cell_edge
cytoplasmic
none
nuclear
nuclear_edge
c
3T3 cells (seqFISH+)
Proportion
transcript count
d
Random Forest
f1score
min_count
pattern
cell_edge
cytoplasmic
none
nuclear
nuclear_edge
e
AUROC Test
0.99
0.98
0.99
0.80
RF
SVM
NN
CNN
AUROC Validation
seqFISH+
0.76
0.70
0.69
0.76
MERFISH
0.81
0.74
0.78
0.49
RF
SVM
NN
CNN

Fig S1. RNAforest performance evaluation. A) Cumulative distribution of sample molecule copy number in U2-OS cells MERFISH dataset. **B)** Validation F1-score of each binary classifier in RNAforest as a function of sample molecule copy number for MERFISH dataset. **C)** Cumulative distribution of sample molecule copy number in 3T3 cells seqFISH+ dataset. **D)** Validation F1-score of each binary classifier in RNAforest as a function of sample molecule copy number for seqFISH+ dataset. **E)** Benchmarking performance of the 4 base models (RF - random forest, SVM - support vector machine, NN - fully connected neural network, CNN - convolutional neural network), showing AUROC in test and validation data.

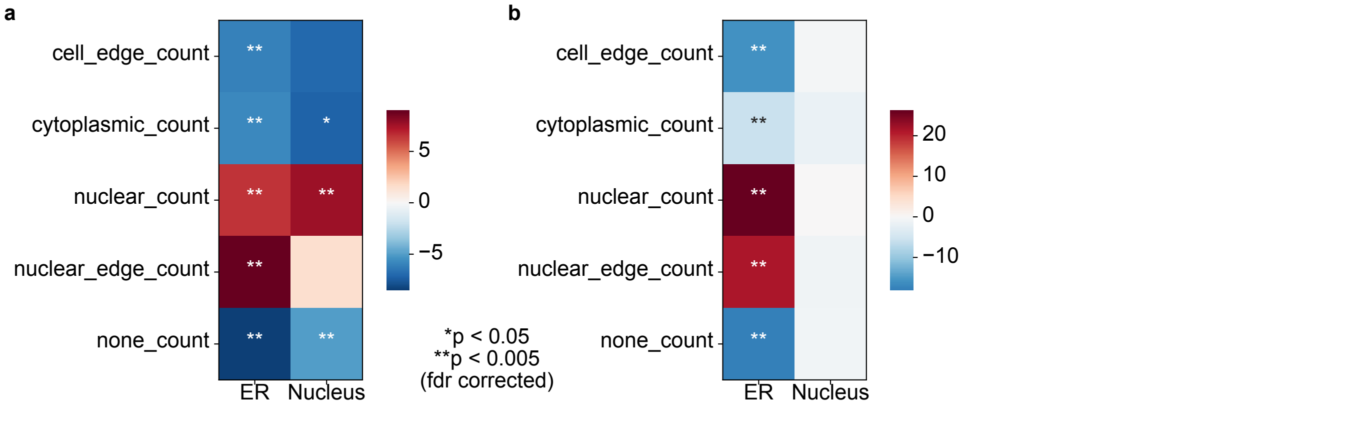
a
cell_edge_count
cytoplasmic_count
nuclear_count
nuclear_edge_count
none_count
ER
Nucleus
**
**
*
**
**
**
**
**
5
0
−5
*p < 0.05
**p < 0.005
(fdr corrected)
b
cell_edge_count
cytoplasmic_count
nuclear_count
nuclear_edge_count
none_count
ER
Nucleus
**
**
**
**
**
20
10
0
−10

Fig S2. Enrichment of compartment-specific expression for RNAforest gene pattern frequencies. Compartment-specific enrichment of endoplasmic reticulum (ER) and nucleus gene expression – from Xia et al 2019[46] – relative to RNAforest gene pattern frequencies in the **A)** MERFISH dataset and **B)** seqFISH+ dataset.

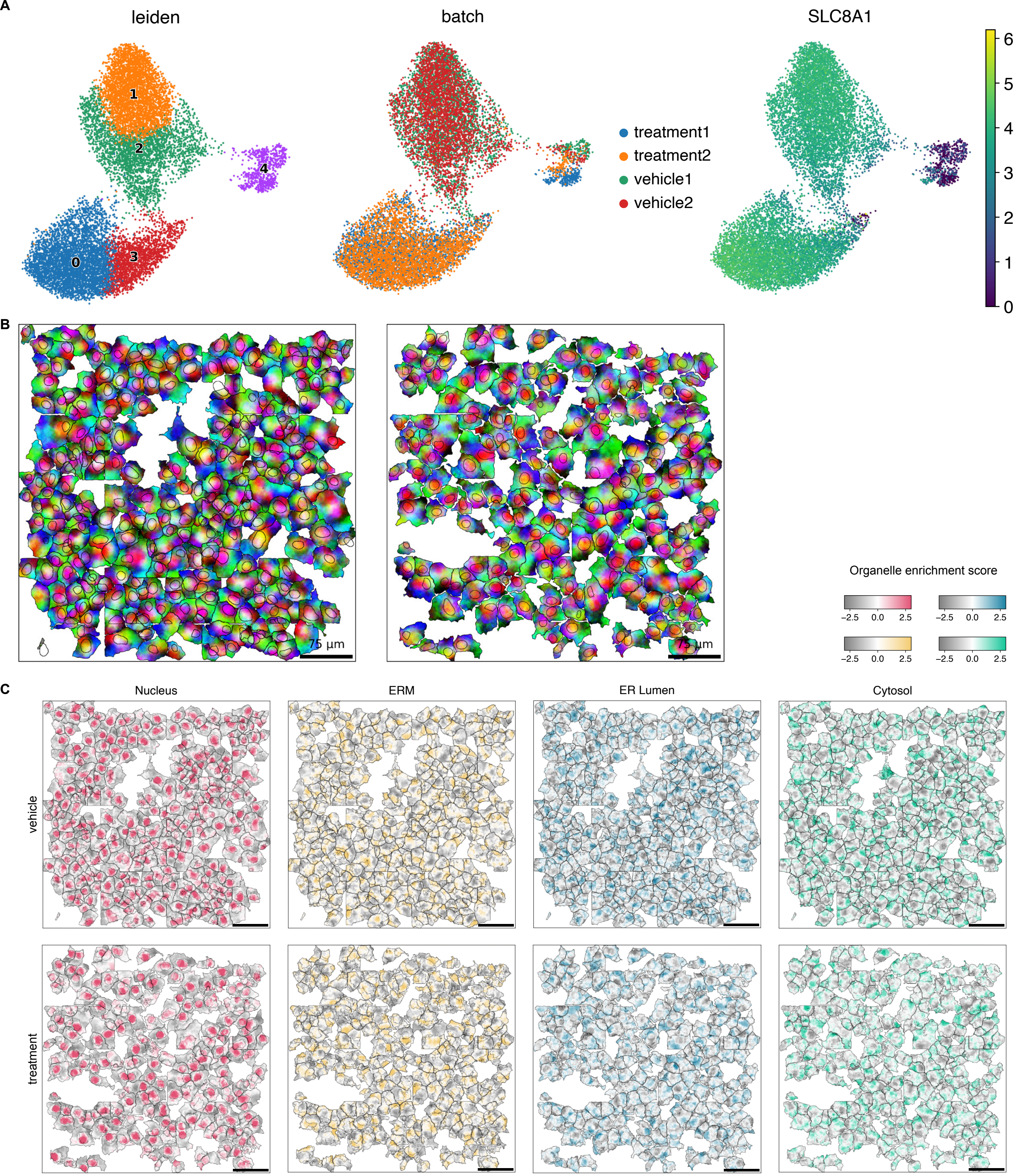
A
leiden
batch
SLC8A1
treatment1
treatment2
vehicle1
vehicle2
0
1
2
3
4
5
6
B
75 µm
75 µm
Organelle enrichment score
−2.5
0.0
2.5
C
Nucleus
ERM
ER Lumen
Cytosol
vehicle
treatment

Fig S3. Filtering and RNAflux analysis of DOX treated cardiomyocytes. A) Left: UMAP of all 4 cardiomyocyte samples. Leiden Clustering identifies 5 clusters, separating low expression SLC8A1 into cluster 4.. Center: Cells are colored to denote different samples. Right: Cells are colored by log scaled SLC8A1 RNA expression. **B)** Representative crop of vehicle (left) and treatment (right) samples, colored by the first 3 principal components of its RNAflux embedding. **C)** Relative enrichment of transcripts enriched for location-specific expression in vehicle (top) and treatment (bottom) samples. Red, yellow, blue and green enrichment correspond to nuclear, ER membrane, ER lumen, and cytosol genesets respectively.

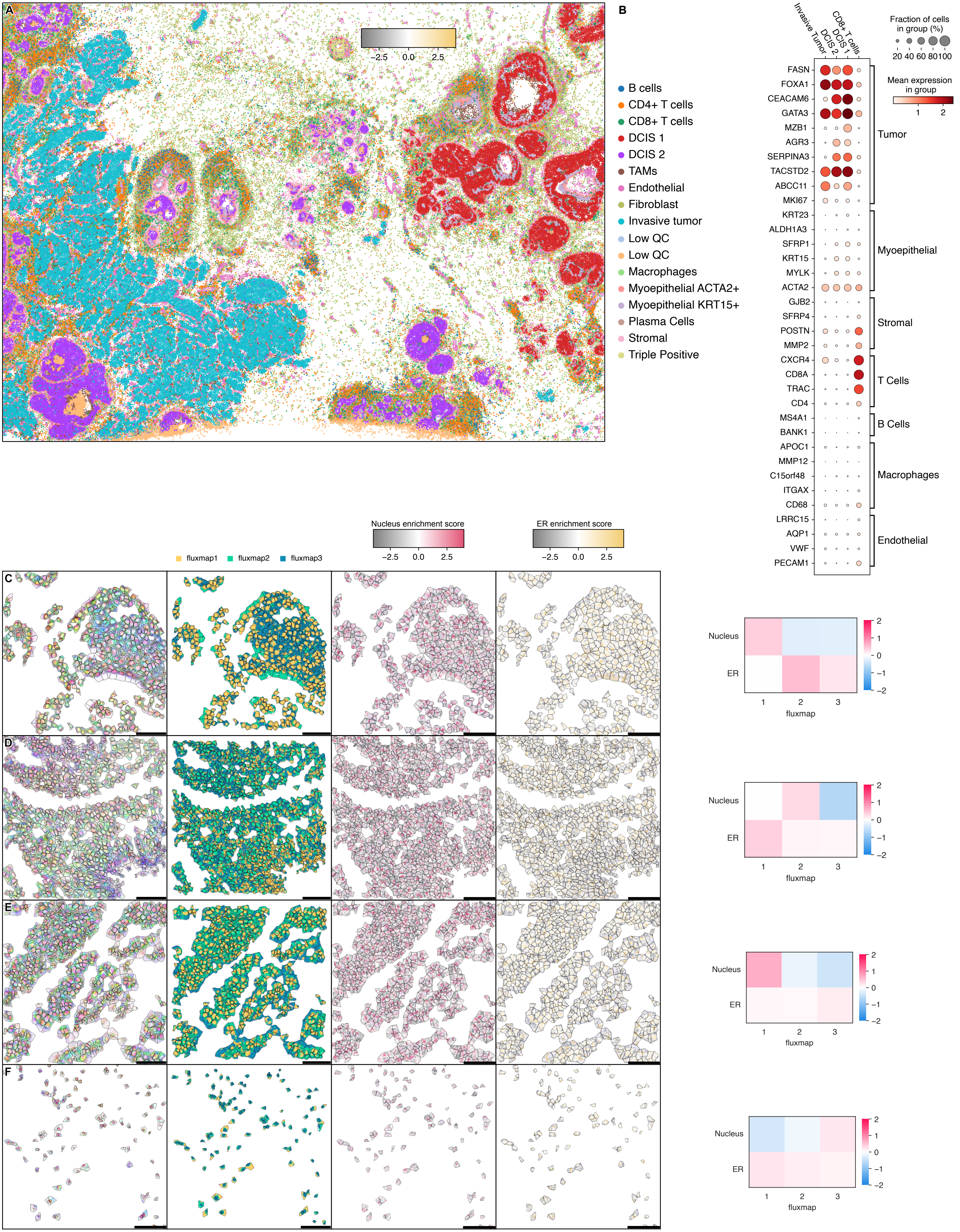
A
B
−2.5 0.0 2.5
B cells
CD4+ T cells
CD8+ T cells
DCIS 1
DCIS 2
TAMs
Endothelial
Fibroblast
Invasive tumor
Low QC
Low QC
Macrophages
Myoepithelial ACTA2+
Myoepithelial KRT15+
Plasma Cells
Stromal
Triple Positive
Invasive Tumor
DCIS 2
DCIS 1
CD8+ T cells
Fraction of cells in group (%)
20 40 60 80 100
Mean expression in group
1 2
FASN
FOXA1
CEACAM6
GATA3
MZB1
AGR3
SERPINA3
TACSTD2
ABCC11
MKI67
Tumor
KRT23
ALDH1A3
SFRP1
KRT15
MYLK
ACTA2
Myoepithelial
GJB2
SFRP4
POSTN
MMP2
Stromal
CXCR4
CD8A
TRAC
CD4
T Cells
MS4A1
BANK1
B Cells
APOC1
MMP12
C15orf48
ITGAX
CD68
Macrophages
LRRC15
AQP1
VWF
PECAM1
Endothelial
fluxmap1
fluxmap2
fluxmap3
Nucleus enrichment score
−2.5 0.0 2.5
ER enrichment score
−2.5 0.0 2.5
C
D
E
F
Nucleus
ER
1 2 3
fluxmap
2 1 0 −1 −2

Fig S4. RNAflux analysis of breast cancer tissue Xenium dataset. A) Cells are colored by cell type identity. **B)** Expression of top marker genes for each cell type. **C) Left:** Visualization of RNAflux results in order of top to bottom: DCIS 1, DCIS 2, invasive tumor, CD8+ T cells. First column presents the RNAflux results by coloring local compositions by their principal components 1-3 weights. Second column shows the fluxmap segmentations with three clusters. Third column shows the enrichment score of nuclear genes in each local composition. The fourth column similarly shows the enrichment scores of endoplasmic reticulum genes in each local composition. **Right:** Summary heatmap of nuclear and ER gene enrichment in the three fluxmaps for each tissue region.

Table S1

	Categories	Features
**1.**	**Distance**	Cell inner proximity: The average distance between all points within the cell to the cell boundary normalized by cell radius. Values closer to 0 denote farther from the cell boundary, values closer to 1 denote closer to the cell boundary.
**2.**	**Distance**	Nucleus inner proximity: The average distance between all points within the nucleus to the nucleus boundary normalized by cell radius. Values closer to 0 denote farther from the nucleus boundary, values closer to 1 denote closer to the nucleus boundary.
**3.**	**Distance**	Nucleus outer proximity: The average distance between all points within the cell and outside the nucleus to the nucleus boundary normalized by cell radius. Values closer to 0 denote farther from the nucleus boundary, values closer to 1 denote closer to the nucleus boundary.
**4.**	**Symmetry**	Cell inner asymmetry: The offset between the centroid of all points within the cell to the centroid of the cell boundary, normalized by cell radius. Values closer to 0 denote symmetry, values closer to 1 denote asymmetry.
**5.**	**Symmetry**	Nucleus inner asymmetry: The offset between the centroid of all points within the nucleus to the centroid of the nucleus boundary, normalized by cell radius. Values closer to 0 denote symmetry, values closer to 1 denote asymmetry.
**6.**	**Symmetry**	Nucleus outer asymmetry: The offset between the centroid of all points within the cell and outside the nucleus to the centroid of the nucleus boundary, normalized by cell radius. Values closer to 0 denote symmetry, values closer to 1 denote asymmetry.
**7.**	**Dispersion**	Point dispersion: The second moment of all points in a cell relative to the centroid of the total RNA signal. This value is normalized by the second moment of a uniform distribution within the cell boundary.
**8.**	**Dispersion**	Nucleus dispersion: The second moment of all points in a cell relative to the centroid of the nucleus boundary. This value is normalized by the second moment of a uniform distribution within the cell boundary.
**9.**	**Density**	*L-function max: The max value of the L-function evaluated at r=[1,d], where d is half the cell's maximum diameter.
**10.**	**Density**	*L-function max gradient: The max value of the gradient of the above L-function.
**11.**	**Density**	*L-function min gradient: The min value of the gradient of the above L-function.
**12.**	**Density**	*L monotony: The correlation of the L-function and r=[1,d].
**13.**	**Density**	*L-function at d/2: The value of the L-function evaluated at ¼ of the maximum cell diameter.

*The L-function measures spatial clustering of a point pattern over an area of interest.
